# Adenylate kinase 4 modulates oxidative stress and stabilizes HIF-1α to drive lung adenocarcinoma metastasis

**DOI:** 10.1186/s13045-019-0698-5

**Published:** 2019-01-29

**Authors:** Yi-Hua Jan, Tsung-Ching Lai, Chih-Jen Yang, Yuan-Feng Lin, Ming-Shyan Huang, Michael Hsiao

**Affiliations:** 1 Genomics Research Center, Academia Sinica, 128 Academia Road, Section 2, Taipei, 115 Taiwan; 2 Division of Pulmonary and Critical Care Medicine, Department of Internal Medicine, Kaohsiung Medical University Hospital, Kaohsiung Medical University, Kaohsiung, Taiwan; 3 Faculty of Medicine, College of Medicine, Kaohsiung Medical University, Kaohsiung, Taiwan; 4 Graduate Institute of Clinical Medicine, College of Medicine, Taipei Medical University, Taipei, Taiwan; 5 Department of Internal Medicine, E-DA Cancer Hospital, School of Medicine, I-Shou University, Kaohsiung, Taiwan; 6 Department of Biochemistry, College of Medicine, Kaohsiung Medical University, Kaohsiung, Taiwan

**Keywords:** AK4, Lung cancer metastasis, ROS, HIF-1α, EMT, Withaferin-A

## Abstract

**Background:**

Adenylate kinase 4 (AK4) has been identified as a biomarker of metastasis in lung cancer. However, the impacts of AK4 on metabolic genes and its translational value for drug repositioning remain unclear.

**Methods:**

Ingenuity upstream analyses were used to identify potential transcription factors that regulate the AK4 metabolic gene signature. The expression of AK4 and its upstream regulators in lung cancer patients was examined via immunohistochemistry. Pharmacological and gene knockdown/overexpression approaches were used to investigate the interplay between AK4 and its upstream regulators during epithelial-to-mesenchymal transition (EMT). Drug candidates that reversed AK4-induced gene expression were identified by querying a connectivity map. Orthotopic xenograft mouse models were established to evaluate the therapeutic efficacy of drug candidates for metastatic lung cancer.

**Results:**

We found that HIF-1α is activated in the AK4 metabolic gene signature. IHC analysis confirmed this positive correlation, and the combination of both predicts worse survival in lung cancer patients. Overexpression of AK4 exaggerates HIF-1α protein expression by increasing intracellular ROS levels and subsequently induces EMT under hypoxia. Attenuation of ROS production with *N*-acetylcysteine abolishes AK4-induced invasion potential under hypoxia. Pharmacogenomics analysis of the AK4 gene signature revealed that withaferin-A could suppress the AK4-HIF-1α signaling axis and serve as a potent anti-metastatic agent in lung cancer.

**Conclusions:**

Overexpression of AK4 promotes lung cancer metastasis by enhancing HIF-1α stability and EMT under hypoxia. Reversing the AK4 gene signature with withaferin-A may serve as a novel therapeutic strategy to treat metastatic lung cancer.

**Electronic supplementary material:**

The online version of this article (10.1186/s13045-019-0698-5) contains supplementary material, which is available to authorized users.

## Introduction

Non-small cell lung cancer (NSCLC) remains the leading cause of cancer-related death around the world, mainly due to its high metastatic rate [1]. Recently, metabolic reprogramming has been considered an important feature that drives malignant progression of tumors [2]. In metastatic cancer cells, energy metabolism is altered due to constant exposure to oxidative stress and chronic nutrient and oxygen depletion. To fulfill biosynthetic and redox requirements, cancer cells consume glucose and secrete lactate even when oxygen is available, a phenomenon known as aerobic glycolysis or the “Warburg effect” [3]. Hypoxia-inducible factor-1α (HIF-1α) is a key transcription factor in the cell response to hypoxic stress. HIF-1α transcribes genes involved in glycolysis, angiogenesis, and cancer metastasis [4, 5]. During metabolic stress, AMP-activated protein kinase (AMPK) is activated by sensing a decrease in the ratio of ATP to AMP, leading to inhibition of ATP-consuming metabolic pathways and activation of energy-producing pathways [6]. In addition, adenylate kinases (AKs), which are abundant nucleotide phosphotransferases, catalyze the generation of two molecules of ADP by transferring a phosphate group from one molecule of ATP or GTP to AMP. The main role of AKs is to balance cellular adenine nucleotide composition to maintain energy homeostasis [7]. However, the link between energy homeostasis and cancer progression has not been clearly elucidated.

Adenylate kinase 4 (AK4) is localized in the mitochondrial matrix [8] and has been shown to physically bind to mitochondrial ADP/ATP translocase (ANT) as a stress-responsive protein to maintain cell survival [9]. Moreover, several genomic and proteomic studies have shown that AK4 expression fluctuates under cellular stress conditions [10–13]. Significantly increased AK4 protein levels have been detected during development, in cultured cells exposed to hypoxia and in an animal model of amyotrophic lateral sclerosis [9, 14–16]. Moreover, Lanning et al. showed that silencing of AK4 elevates the cellular ATP level up to 25% and concurrently increases the ADP/ATP ratio, which activates AMPK signaling [17]. Previously, we identified AK4 as a lung cancer progression marker by assessing the correlation between AK4 levels and clinicopathological features [18]. However, how AK4-induced metabolic changes may affect cancer progression remains unclear.

Here, we aimed to investigate the impact of AK4 expression on metabolic genes by analyzing lung cancer microarray datasets and decipher the functional consequences on lung cancer metastasis. We found that HIF-1α activity is significantly activated in lung adenocarcinoma patients with an AK4 metabolic gene signature. Overexpression of AK4 shifts metabolism toward aerobic glycolysis and increases the levels of intracellular reactive oxygen species (ROS), which subsequently stabilizes HIF-1α protein and promotes epithelial-to-mesenchymal transition (EMT) in lung cancer cells in a HIF-1α-dependent manner. These findings represent a novel vicious cycle between AK4 and HIF-1α in response to hypoxic stress during lung cancer progression and highlight the therapeutic opportunity of targeting the AK4-HIF-1α axis in NSCLC.

## Materials and methods

### Specimens

Clinical non-small cell lung cancer (NSCLC) samples were collected with IRB approval (KMUHIRB-E(I)-20160099) from the Kaohsiung Medical University Hospital and were fixed in formalin and embedded in paraffin before being archived. The archived specimens, with follow-up times up to 200 months, were used for immunohistochemical staining. The histologic diagnosis was made according to the World Health Organization (WHO) classification guidelines for lung cancer. The pathological diagnosis of tumor size, local invasion, lymph node involvement, distal metastasis, and final disease stage were determined according to the American Joint Committee on Cancer (AJCC) TNM classification of lung cancer.

### Tissue microarray and immunohistochemical staining

Representative 1-mm-diameter cores from each tumor sample were selected by matching histology from original hematoxylin and eosin (H&E)-stained slides, and the histopathologic diagnosis of all samples was reviewed and confirmed by pathologists. IHC staining was performed using an automated immunostainer (Ventana Discovery XT autostainer, Ventana, USA) with a 30-min heat-induced antigen retrieval procedure in TRIS-EDTA buffer. Protein expression was visualized using a 3,3′-diaminobenzidine (DAB) peroxidase substrate kit (Ventana, USA). The following antibodies were used to detect AK4, HIF-1α, E-cadherin, and pimonidazole in tissues: AK4 (Genetex, 1:200), HIF-1α (Cell Signaling, 1:100), E-cadherin (Cell Signaling, 1:100), and pimonidazole (Hypoxyprobe, INC).

### Histology and IHC staining interpretation

The IHC staining results were assessed and scored independently by two pathologists who were blinded to the patient clinical outcomes. A consensus decision was made when there was an interobserver discrepancy. For scoring, both intensity and percentage of protein expression were recorded. The staining intensity was scored as follows: 0, no staining; 1+, weak staining; 2+, moderate staining; 3+, strong staining. The extent of staining was further divided into two groups according to 25% of tumor cells with staining. A high IHC expression level was defined as a staining intensity of 2+ or 3+ in over 25% of tumor cells.

### Microarray data analysis

The raw intensities of AK4 overexpression in CL1-0 cells (GSE37903) and lung adenocarcinoma patient datasets (GSE31210) were normalized by robust multichip analysis (RMA) using GeneSpring GX11 (Agilent Technologies). AK4-associated gene signatures were identified by calculating the Pearson correlation coefficient between AK4 expression and each coding gene and ranked according to their correlation coefficient to AK4 expression. After applying a Pearson correlation coefficient of ± 0.3 as a threshold, the AK4 metabolic gene signature was identified by selecting genes with enzyme or transporter annotations. Next, gene set enrichment analysis (GSEA) was performed to rank the probes and analyze gene set enrichment using c2.all.v5.1.symbols.gmt [curated] or c2.cp.kegg.v5.1.symbols.gmt [curated] gene sets as a backend database (http://www.broadinstitute.org/gsea). *P* values less than 0.05 and FDRs less than 25% were considered to indicate significant enrichment.

The activation or inhibition status of upstream regulators in the AK4 metabolic gene signature was predicted using IPA Upstream Regulator Analysis (Ingenuity Systems, http://www.ingenuity.com), and the calculated *z* scores can reflect the overall activation state of the regulator (< 0: inhibited, > 0: activated). In practice, a *z* score of more than 2 or less than − 2 can be considered significant activation or inhibition, respectively.

### Cell lines

The human lung adenocarcinoma cell lines H1355, PC9, H358, H928, CL1-0 CL1-1, CL1-3, and CL1-5 and squamous cell carcinoma cell lines H157 and H520 were maintained in RPMI 1640 medium (Invitrogen) supplemented with 10% fetal bovine serum (FBS). Human lung adenocarcinoma cell lines (A549, PC13, and PC14) and large cell carcinoma H1299 cells were grown in DMEM (Invitrogen) containing 10% FBS. All cells were kept under a humidified atmosphere containing 5% CO_2_ at 37 °C. CL1-0, CL1-1, CL1-3, and CL1-5 cell lines were established by Chu et al. at National Taiwan University Hospital and displayed progressively increased invasiveness, while PC13 and PC14 cell lines were derived from Tokyo National Cancer Centre Hospital. Other lung cancer cell lines (A549, H1355, H358, H928, H520, H157, H460, and H1299) were obtained from American Type Culture Collection.

### Lentiviral shRNA and expression vectors

GIPZ Lentiviral AK4 (AK3L1) shRNA and HIF1A shRNA constructs, which carry the puromycin resistance gene and enhanced green fluorescent protein (EGFP), were purchased from Open Biosystems. Lentiviruses were generated by transfecting 293 T cells with the shRNA-expression vector and pMD2.G and pDeltaR8.9 using the calcium phosphate precipitation method. Virus-containing supernatants were collected, titrated, and used to infect cells using 8 μg/mL polybrene. Infected cells were selected using 2 μg/mL puromycin. For expression of AK4, full-length AK4 cDNA was cloned into a pLenti6.3 lentiviral vector (Invitrogen). AK4-expressing cell lines were established by infecting cells with the pLenti6.3-AK4 viruses generated by transfection of 293 T cells with pLenti6.3 AK4, pMD2.G, and pDeltaR8.91. Cells were then selected in 5 μg/mL blasticidin.

### Western blot analysis

The following antibodies were used in western blot analyses: anti-AK4 (Genetex, 1:2000), anti-HIF-1α (Cell Signaling, 1:1000), anti-hydroxylated HIF-1α (Cell Signaling, 1:1000), anti-E-cadherin (BD Bioscience, 1:1000), anti-vimentin (Sigma, 1:2000), anti-Snail (Cell Signaling, 1:1000), and anti-α-tubulin (Sigma-Aldrich, 1:5000) antibodies.

### Reagent and chemicals

Proscillaridin, ouabain, digitoxigenin, digoxin, withaferin-A, and lanatoside-C were purchased from Sigma-Aldrich (St. Louis, MO). ATP colorimetric assay, glucose colorimetric assay, and lactate colorimetric assay kits were purchased from BioVision (Milpitas, CA). CellROX Deep Red Reagent was purchased from Invitrogen.

### Cycloheximide assay

Cells were plated in 6-well plates and treated with cycloheximide (CHX) at a concentration of 50 μg/mL for 24 h. Cells were then exposed to hypoxia for 6 h to stabilize HIF-1α protein and then switched to normoxic conditions. Protein lysates were harvested at 20-min intervals under normoxic conditions.

### ATP measurement

Cells were grown in a 6-well plate overnight, and the medium was refreshed with complete medium. After 24 h, a cell pellet was collected, and the amount of ATP was quantified using an ATP colorimetric assay kit (BioVision) according to the manufacturer’s protocol.

### Glucose consumption assay

Cells were grown in a 6-well plate overnight, and the medium was refreshed with complete medium. After 24 h, the spent medium was collected, and the amount of glucose was quantified using a glucose colorimetric assay kit (BioVision) according to the manufacturer’s protocol.

### Lactate production assay

Cells were grown in a 6-well plate overnight, and the medium was refreshed with complete medium. After 24 h, the spent medium was collected, and the amount of lactate was quantified using a lactate colorimetric assay kit (BioVision) according to the manufacturer’s protocol.

### ROS measurement

ROS levels were quantified using CellROX Deep Red Reagent (Invitrogen). Briefly, cells were seeded in a 96-well plate (2000 cells/well) and washed with PBS. Cells were then incubated with 5 μM CellROX for 30 min at 37 °C and stained with DAPI. Intracellular ROS were measured using a fluorescence plate reader at absorption/emission wavelengths of ~ 644/665 nm.

### Invasion assay

Polycarbonate filters were coated with human fibronectin on the lower side and Matrigel on the upper side. Medium containing 10% FCS was added to each well of the lower compartment of the chamber. Cells were suspended in serum-free medium containing 0.1% bovine serum albumin and loaded into each well of the upper chamber. After 16 h, cells were fixed with methanol and then stained with Giemsa. Cells that invaded to the lower side of the membrane were counted under a light microscope (× 200, ten random fields in each well). All experiments were performed in quadruplicate.

### Animal studies

All animal experiments were conducted according to protocols approved by the Academia Sinica Institutional Animal Care and Unitization Committee. Age-matched NOD-SCID Gamma (NSG) mice (6–8 weeks old) were used to construct xenograft models. For the subcutaneous xenograft model, cells were subcutaneously injected into the flanks of NSG mice at a concentration of 1 × 10^6^ cells in 100 μL of PBS. Tumor volumes were measured weekly for 4 weeks. At the endpoint, mice were intravenously injected with Hypoxyprobe™ (Hypoxyprobe, Inc) solution at a dosage of 60 mg/kg body weight and tumors were removed and analyzed for hypoxic necrosis using immunostaining of pimonidazole adducts in tissues. Slides were digitally scanned using ScanScope AT (Aperio Technologies Inc.). Quantification of hypoxic and non-hypoxic areas in pimonidazole-stained slides was performed using Definiens’ Tissue Studio software (Definiens Inc.). For the orthotopic xenograft model of lung cancer metastasis, 1 × 10^5^ CL1-0 Vec cells, CL1-0 AK4 cells, or A549-GL cells (established by infecting cells with EF1 promoter-driven firefly luciferase viruses and IRES-driven EGFR viruses) were suspended in 10 μl of PBS/Matrigel mixture (1:1) and injected into the left lung of NSG mice (*n* = 6 per group). Mice from the withaferin-A treatment group were administered 1 mg/kg body weight or 4 mg/kg body weight withaferin-A in 100 μL of PBS three times per week via i.p. injection. Control mice were injected with 100 μL of vehicle PBS containing less than 10% DMSO. Four weeks postinjection, mice were sacrificed, and metastatic liver nodules were counted by gross examination. H&E staining was performed to confirm the histology of metastatic nodules.

### Statistical analysis

Statistical analyses were performed using SPSS 17.0 software (SPSS, USA). The correlation between AK4 and HIF-1α expression determined via IHC was assessed using Spearman’s rank correlation analysis. Estimates of survival rates were obtained using the Kaplan-Meier method and compared with a log-rank test. For all analyses, a *P* value < 0.05 was considered statistically significant. All observations were confirmed in at least three independent experiments. The results are presented as the mean ± SD. We used two-tailed, unpaired Student’s *t* tests for all pairwise comparisons.

## Results

### The transcription factor HIF-1α is active in the AK4 metabolic gene signature in lung adenocarcinoma patients

**Fig. 1:**
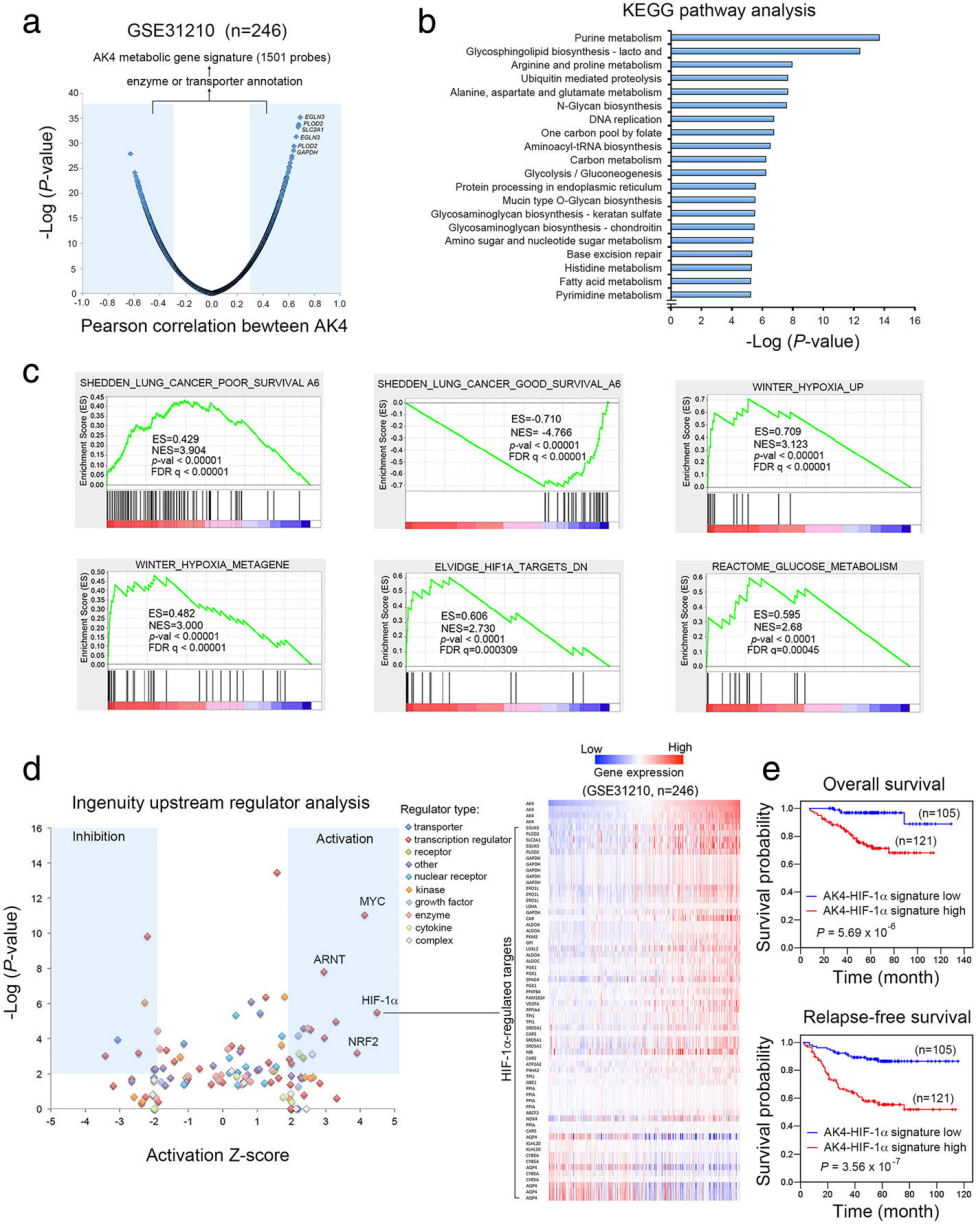
Upstream analysis of the AK4 metabolic gene signature predicted that HIF-1α is transcriptionally activated in lung adenocarcinomas. **a** Genes were ranked according to their corresponding Pearson correlation coefficient (*R*) to AK4 expression. Genes that are positively correlated with AK4 (*R* ≥ 0.3) or negatively correlated with AK4 (*R* ≤ − 0.3) were further filtered with enzyme or transporter annotations and defined as the AK4 metabolic gene signature. **b** KEGG pathway analysis of the AK4 metabolic gene signature. Bar chart represents top 20 significant metabolic pathways ranked according to – log enrichment *P* value. *P* values were calculated using Fisher exact test. **c** GSEA plots of lung cancer prognostic, hypoxic response, and glucose metabolism genes in the AK4 metabolic gene signature. **d** Left, the ingenuity upstream regulator analysis algorithm predicted significant activation or inhibition of upstream regulators in the AK4 metabolic gene signature. An activation *z* score of more than 2 or less than − 2 was considered to indicate significant activation or inhibition, respectively. Right, a heatmap illustrating both direct and indirect HIF-1α-regulated genes that are positively or negatively correlated with AK4 expression. **e** Left, overall survival analysis of patients stratified according to the AK4-HIF-1α gene expression signature. Right, relapse-free survival analysis of patients stratified according to the AK4-HIF-1α gene expression signature

To identify the AK4 metabolic gene signature, we analyzed a lung cancer dataset (GSE31210) that contained microarray data from 246 stage I/II lung adenocarcinoma patients. We first calculated the Pearson correlation coefficient between AK4 expression and each coding gene and ranked the genes according to their correlation coefficient to AK4 expression. We then determined the AK4 metabolic gene signature by selecting genes annotated with enzyme or transporter and applying ± 0.3 Pearson correlation coefficient as the cutoff threshold. By this means, we identified 1501 probes that were significantly associated with AK4 expression in lung adenocarcinoma patients (Fig. 1a). KEGG pathway analysis further revealed top 20 enriched metabolic pathways in the AK4 metabolic gene signature (Fig. 1b). Next, we subjected the AK4 metabolic gene signature to gene set enrichment analysis (GSEA). Interestingly, the GSEA results showed that gene sets categorized as hypoxia response, HIF1A target, glucose metabolism, and lung cancer prognostic genes were significantly enriched in the AK4 metabolic gene signature (Fig. 1c). Furthermore, ingenuity upstream regulator analysis showed that HIF-1α was ranked as the top activated transcription factor, with an activation *z* score of 4.3 (Fig. 1d, left). A heatmap illustrated both direct and indirect HIF-1α-regulated genes that were positively or negatively correlated with AK4 expression (Fig. 1d, right). Kaplan-Meier survival analysis showed that high expression of the AK4-HIF-1α signature in patients was significantly associated with worse overall and relapse-free survival compared with low levels of the AK4-HIF-1α signature (Fig. 1e). Meanwhile, we also identified the consensus AK4 metabolic gene signature between the GSE31210 lung adenocarcinoma dataset and TCGA LUAD dataset and found that HIF-1α was again significantly activated, with an activation *z* score of 4.098 (Additional file 1: Figure S1).

### Combined AK4 and HIF-1α expression predicts a worse prognosis compared with HIF-1 α alone in NSCLC patients

**Fig. 2:**
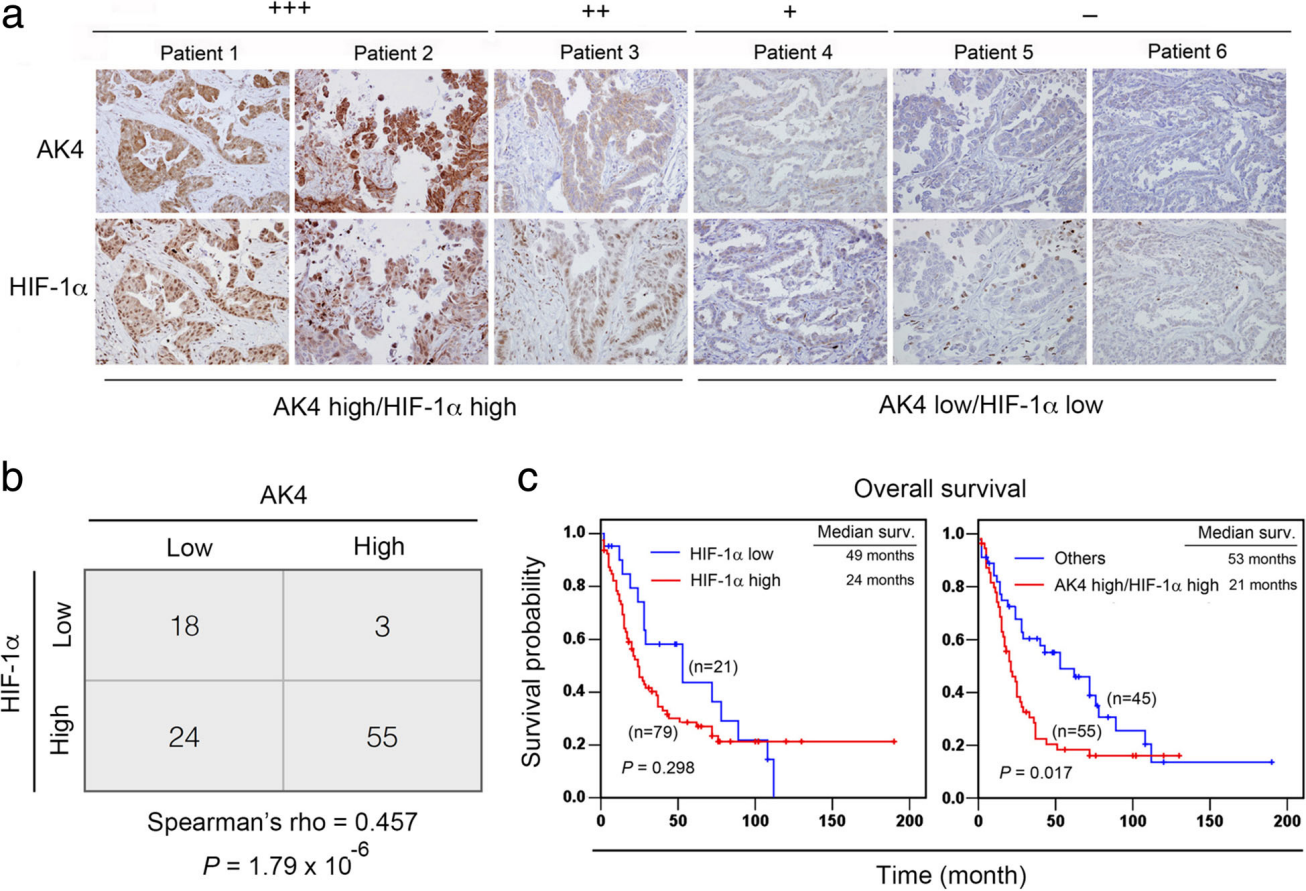
AK4 is positively correlated with HIF-1α expression in NSCLC patients. **a** Representative IHC images of AK4 and HIF-1α expression scores in serial sections of NSCLC tissues. **b** Spearman’s rho correlation analysis of the IHC staining results for AK4 and HIF-1α in 100 NSCLC patients. **c** Overall survival analysis of 100 lung cancer patients stratified according to HIF-1α alone or both AK4 and HIF-1α

To validate the correlation between AK4 and HIF-1α in clinical specimens, we assessed their expression in 100 NSCLC patients via immunohistochemistry (IHC). Figure 2a shows the scoring criteria for quantifying the expression of AK4 and HIF-1α via immunoreactivity in serial sections. Spearman correlation analysis of the IHC results showed a significant positive correlation between AK4 and HIF-1α (Fig. 2b, Spearman’s rho = 0.457, *P* = 1.79 × 10^− 6^). The associations among AK4 IHC expression, HIF-1α IHC expression, and clinicopathological characteristics including age, gender, smoking history, tumor histology, TNM stage, pathological stage, and tumor recurrence status are summarized in Additional file 1: Table S1. Chi-square analysis showed high expression of AK4 and HIF-1α was significantly associated lymph node involvement (Additional file 1: Table S1). We next investigated the prognostic value of HIF-1α via IHC analysis, and the results showed that patients with high HIF-1α expression tend to have worse survival compared with patients with low HIF-1α expression (Fig. 2c, left, *P* = 0.298). Furthermore, the combined AK4 and HIF-1α status further revealed that patients with high AK4 and high HIF-1α levels exhibited significantly worse outcomes than other patients in this cohort (Fig. 2c, right, *P* = 0.017).

### AK4 exaggerates HIF-1α protein expression under hypoxia and induces EMT

**Fig. 3:**
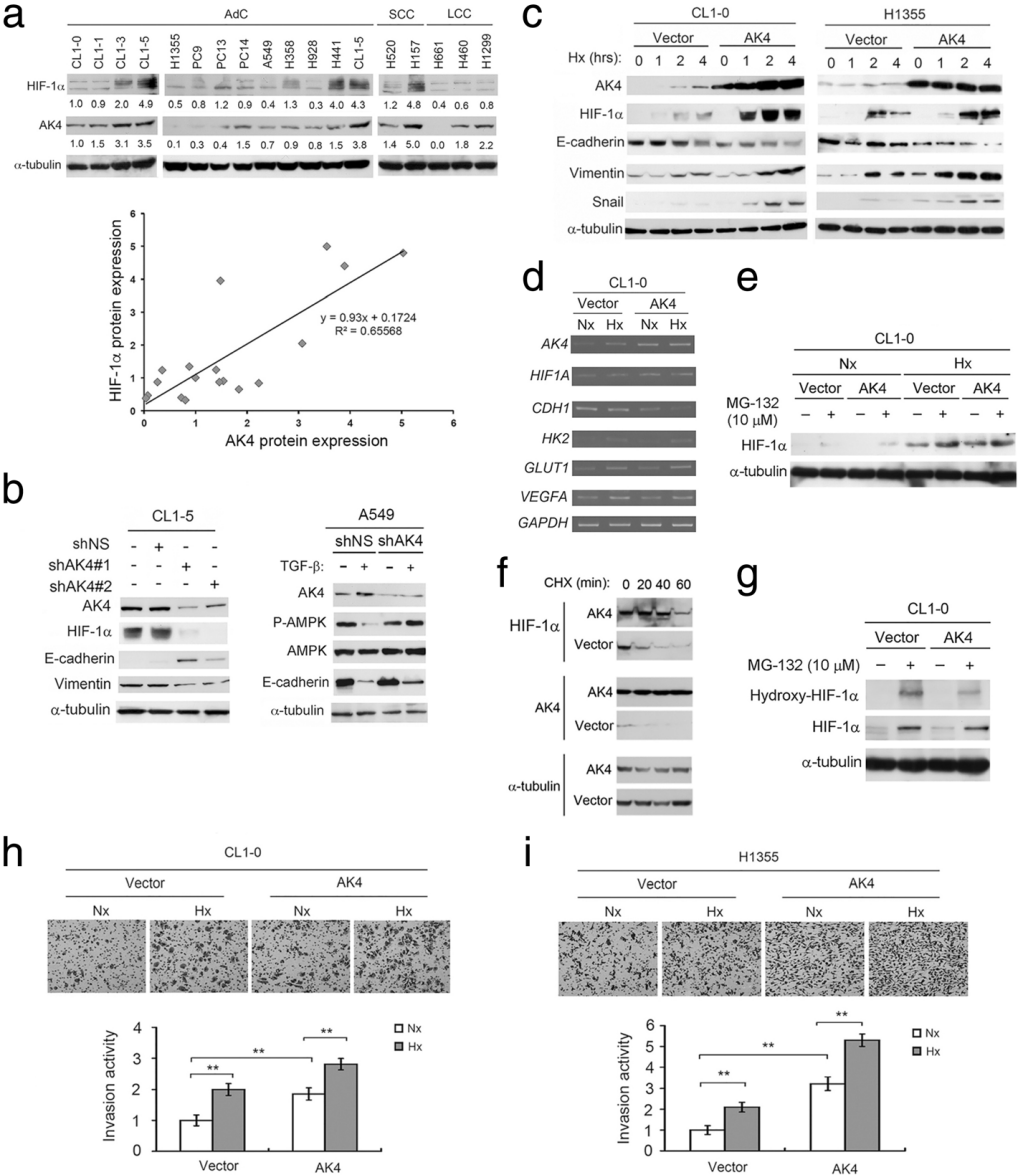
AK4 stabilizes and exaggerates HIF-1α protein expression to promote EMT. **a** Upper, endogenous AK4 and HIF-1α protein expression in human NSCLC cell lines. Bottom, correlation between AK4 and HIF-1α protein expression in NSCLC cell lines. **b** Left, WB analysis of AK4, HIF-1α, E-cadherin, and vimentin upon AK4 knockdown in CL1-5 cells. Right, WB analysis of AK4, AMPK, phospho-AMPK (Thr172), and E-cadherin upon AK4 knockdown in A549 cells treated with or without TGF-β (5 ng/mL) for 24 h. **c** WB analysis of HIF-1α, AK4, E-cadherin, vimentin, and Snail in CL1-0 and H1355 vector- or AK4-overexpressing cells exposed to hypoxia for the indicated time. **d** RT-PCR analysis of *AK4*, *HIF1A*, *CDH1*, *HK2*, *GLUT1*, *VEGFA*, and *GAPDH* in CL1-0 vector- or AK4-expressing cells under normoxic and hypoxic conditions. **e** WB analysis of HIF-1α from CL1-0 vector- or AK4-expressing cells treated with the proteasome inhibitor MG-132 under Nx and Hx conditions. **f** WB analysis of HIF-1α and AK4 from CL1-0 vector- or AK4-expressing cells treated with CHX for 20, 40, and 60 min. **g** WB analysis of HIF-1α and hydroxylated HIF-1α from CL1-0 vector or AK4-expressing cells treated with MG-132 under Nx. **h** Invasion assay of CL1-0 vector- or AK4-expressing cells under normoxia (Nx) or hypoxia (Hx). **P ≤ 0.01. **i** Invasion assay of H1355 vector- or AK4-expressing cells under Nx or Hx. **P ≤ 0.01. The results are presented as the mean ± SD of at least three separate experiments. Two-tailed, unpaired Student’s *t* tests were used for all pairwise comparisons. **P ≤ 0.01

Next, we detected endogenous AK4 and HIF-1α protein expression in 17 human NSCLC cell lines and found that CL1-5, H441, H157, and CL1-3 cells expressed high levels of HIF-1α under normoxic conditions (95% air, 5% CO_2_) and those cells also expressed high levels of AK4 (Fig. 3a, upper). Correlation analysis showed a significant positive correlation between AK4 and HIF-1α (Fig. 3a, bottom). To assess the interaction between AK4 and HIF-1α, we knocked down endogenous AK4 expression using two AK4 shRNA (shAK4) clones in CL1-5 cells. Interestingly, knockdown of AK4 suppressed HIF-1α expression with concurrent upregulation of E-cadherin and downregulation of vimentin (Fig. 3b, left). In a cell model of TGF-β-induced EMT, we found that knockdown of AK4 partially hampered TGF-β-induced EMT with concurrent upregulation of phosphorylated AMPK at Thr172 (Fig. 3b, right). On the other hand, we overexpressed AK4 in CL1-0 cells and H1355 cells and exposed the cells to hypoxic conditions (1% O_2_, 5% CO_2_, 94% N_2_) for various periods of time. Western blot analysis showed that HIF-1α was upregulated earlier and to a higher degree in AK4-overexpressing cells than in vector-expressing cells and concurrently induced EMT in CL1-0 and H1355 cells (Fig. 3c). We next investigated how AK4 regulates HIF-1α protein in CL1-0 cells. RT-PCR analysis showed that AK4 along with several HIF-1α downstream targets, including VEGF, GLUT1, and HK2, were induced upon hypoxia. However, HIF-1α mRNA was not affected by AK4 overexpression (Fig. 3d). Therefore, we hypothesized that the elevation of HIF-1α may be the result of translational control and/or posttranslational regulation. We treated vector- and AK4-overexpressing CL1-0 cells with the proteasome inhibitor MG-132 to block protein degradation and detected HIF-1α protein under normoxia and hypoxia and found that the effect of AK4 overexpression on HIF-1α protein elevation was diminished when the proteasome was inhibited (Fig. 3e). To test whether the enhancement of HIF-1α by AK4 occurs through protein stabilization, we exposed the vector- and AK4-overexpressing CL1-0 cells to hypoxia to induce HIF-1α protein and then treated the cells with cycloheximide (CHX) to block de novo protein synthesis and assessed HIF-1α protein levels at 20-min intervals under normoxic conditions. We found that HIF-1α protein was significantly stabilized in the AK4-overexpressing cells compared with the vector control cells (Fig. 3f). Moreover, we also found that the AK4-overexpressing cells had less hydroxylated HIF-1α, indicating that the prolyl hydroxylase (PHD) activity is lower in AK4-overexpressing cells (Fig. 3g). Moreover, overexpression of AK4 promoted CL1-0 and H1355 cell invasion activity under hypoxia (Fig. 3h, i). To test whether HIF-1α plays a critical role in AK4-induced EMT and invasion activity, we inhibited HIF-1α expression with shRNA in AK4-overexpressing and vector-expressing CL1-0 cells. Knockdown of HIF-1α in AK4-overexpressing cells abolished EMT and suppressed AK4-induced invasion activity under hypoxia (Additional file 1: Figure S2).

### AK4 elevates intracellular ROS levels and promotes aerobic glycolysis

**Fig. 4:**
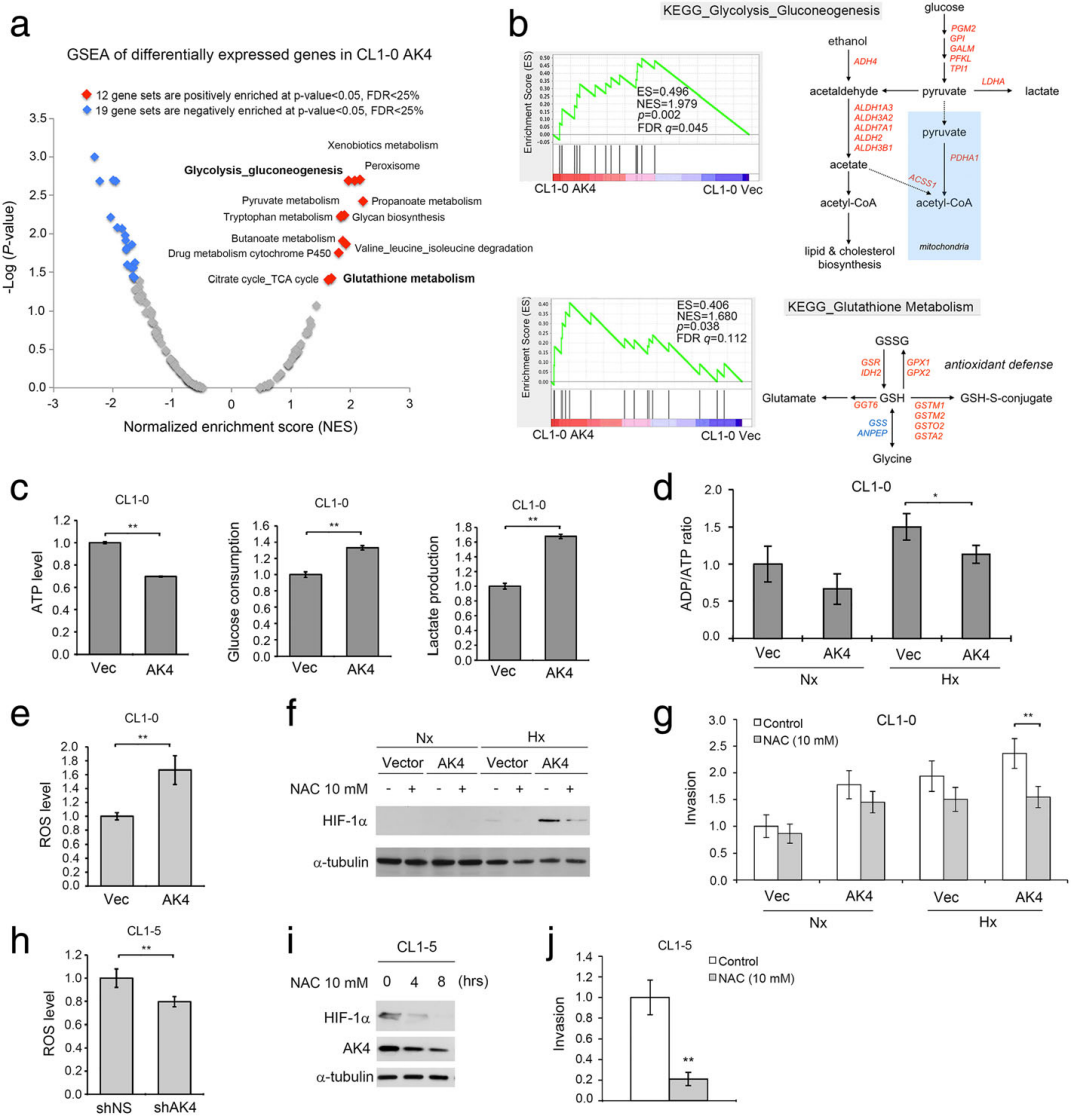
AK4 shifts metabolism toward aerobic glycolysis and increases oxidative stress. **a** Global GSEA statistics of differentially expressed genes in AK4-overexpressing CL1-0 cells compared with vector-expressing CL1-0 cells. **b** GSEA plots and KEGG metabolic pathways of Glycolysis_Gluconeogenesis and Glutathione_Metabolism pathways between CL1-0 AK4 cells and CL1-0 Vec cells. **c** Relative ATP levels, glucose consumption, and lactate production upon AK4 overexpression in CL1-0 cells. **P ≤ 0.01 **d** Relative ADP/ATP ratio upon AK4 overexpression in CL1-0 cells under Nx or Hx. *P ≤ 0.05 **e** Intracellular ROS level of vector-expressing CL1-0 cells and AK4-expressing CL1-0 cells; the ROS level was normalized to vector-expressing CL1-0 cells. **P ≤ 0.01. **f** WB analysis of HIF-1α in CL1-0 vector- and AK4-expressing cells treated with or without 10 mM NAC under Nx or Hx. **g** Invasion assay of CL1-0 vector- or AK4-expressing cells treated with 10 mM NAC under Nx or Hx. **h** Intracellular ROS level of shNS-expressing CL1-5 cells and shAK4-expressing CL1-5 cells. The ROS level was normalized to shNS-expressing CL1-5 cells. **P ≤ 0.01. **i** Time course analysis of HIF-1α and AK4 protein expression in CL1-5 cells treated with 10 mM NAC for the indicated time. **j** Invasion assay of CL1-5 cells treated with or without 10 mM NAC. The results are presented as the mean ± SD of at least three separate experiments. Two-tailed, unpaired Student’s *t* tests were used for all pairwise comparisons. *P ≤ 0.05; **P ≤ 0.01

To decipher the possible metabolic pathways affected by AK4, GSEA was performed to analyze differentially expressed genes in the GSE37930 microarray data (1.5-fold change) in CL1-0 AK4 cells versus CL1-0 Vec cells. We found that 12 KEGG gene sets were positively enriched at the threshold of *P* value < 0.05 and FDR < 25%. The enriched gene sets positively correlated with AK4 expression could be further categorized into four super metabolic pathways: carbohydrate, amino acid, xenobiotic, and oxidative stress pathways (Fig. 4a). Notably, upregulation of AK4 was significantly correlated with genes in glycolysis_gluconeogenesis metabolism and glutathione metabolism (Fig. 4b and Additional file 1: Figure S3). These data prompted us to investigate the effect of AK4 overexpression on glycolysis and oxidative stress. By measuring the levels of ATP, glucose, and lactate, we found that overexpression of AK4 resulted in increased ATP and glucose consumption and increased lactate production (Fig. 4c). Moreover, overexpression of AK4 significantly decreased the ADP/ATP ratio compared with that in control cells upon hypoxia (Fig. 4d). To quantify ROS levels within the cell, we used CellROX deep red to probe intracellular ROS levels in AK4-expressing and vector-expressing CL1-0 cells. The results showed that overexpression of AK4 increased ROS levels 1.67-fold compared with levels in control cells (Fig. 4e). Furthermore, treatment with the antioxidant *N*-acetylcysteine (NAC) abolished AK4-induced stabilization of HIF-1α and invasion under hypoxia (Fig. 4f, g). On the other hand, knockdown of AK4 in CL1-5 cells reduced ROS levels nearly 20% compared with those in shNS control cells (Fig. 4h). NAC treatment in CL1-5 cells not only decreased HIF-1α and AK4 protein expression in a time-dependent fashion but also inhibited cell invasion activity (Fig. 4i, j).

### AK4 reduces hypoxic necrosis and promotes metastasis in vivo

**Fig. 5:**
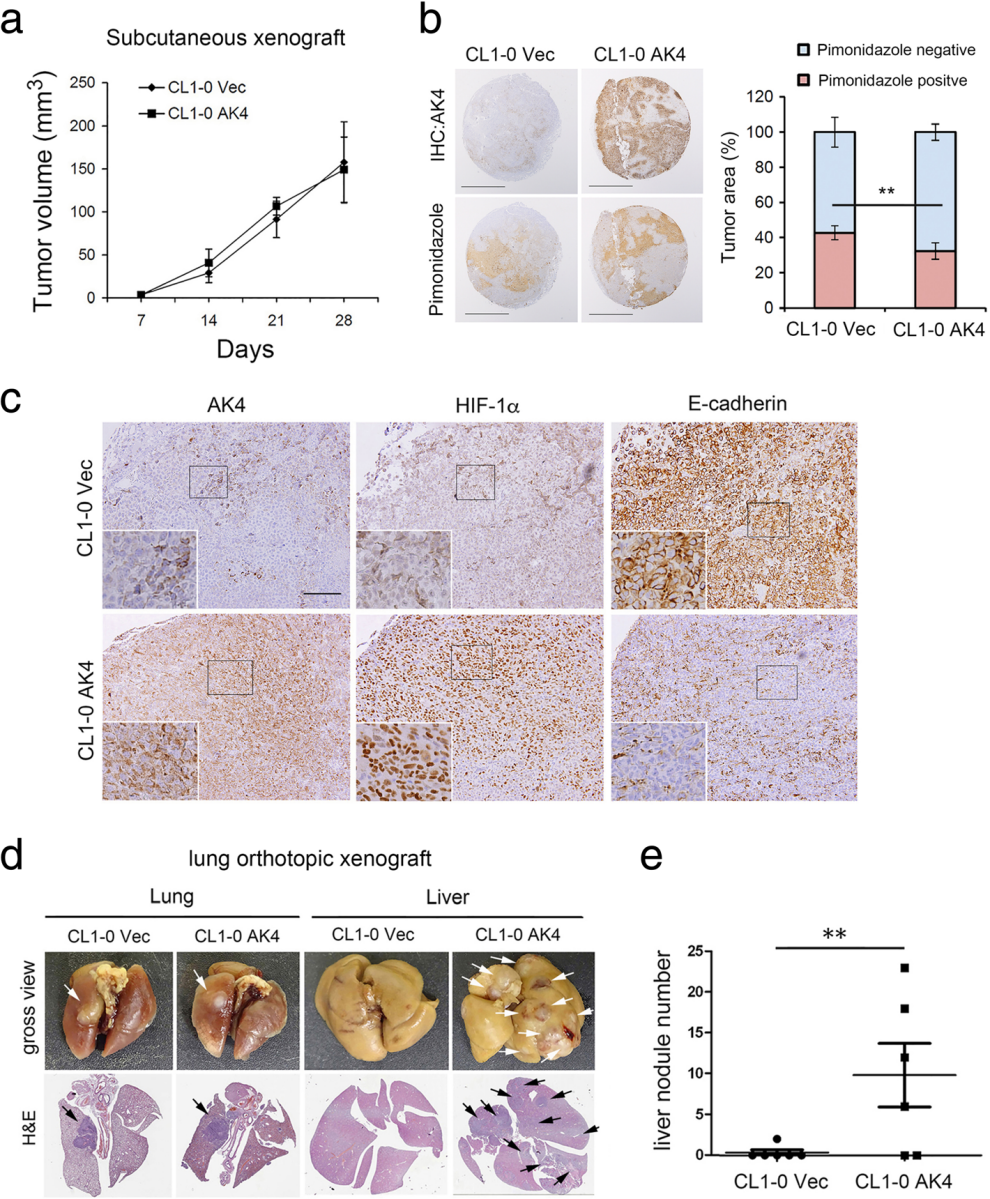
AK4 overexpression reduces hypoxic necrosis and promotes liver metastasis in vivo. **a** NOD scid Gamma (NSG) mice were injected subcutaneously with CL1-0 Vec and CL1-0 AK4 cells (1 × 10^6^ cells/100 μL) in the left and right flanks, respectively. Volumes of CL1-0 Vec and CL1-0 AK4 tumors were measured weekly as indicated. **b** Left, pimonidazole staining and IHC images of AK4 expression in CL1-0 Vec and CL1-0 AK4 subcutaneous xenograft tumors. Scale bar represents 2 mm. Right, pimonidazole-positive tumor area was detected and quantified by Definiens imaging analysis algorithm. **P ≤ 0.01. **c** Representative IHC staining for AK4, HIF-1α, and E-cadherin expression in subcutaneous xenograft tumors from CL1-0 vector or CL1-0 AK4 cells. Scale bar represents 100 μm. **d** NSG mice were injected orthotopically in the left lung with CL1-0 Vec or CL1-0 AK4 cells at a concentration of 1 × 10^5^ cells in 10 μL of PBS/Matrigel mixture. Gross view (formalin-fixed) and H&E staining images of lungs and livers from mice orthotopically injected with CL1-0 Vec or CL1-0 AK4 cells at day 30. The white and black arrows indicate tumor nodules in the gross view and H&E staining images, respectively. **e** Quantification of liver nodule number in mice orthotopically injected with CL1-0 Vec or CL1-0 AK4 cells at day 30. The results are presented as the mean ± SD of at least three separate experiments. Two-tailed, unpaired Student’s *t* tests were used for all pairwise comparisons. *P ≤ 0.05; **P ≤ 0.01

We next investigated the effects of AK4 overexpression on tumorigenicity and metastasis in vivo. CL1-0 vector- and AK4-expressing cells were subcutaneously injected into NSG mice. Four weeks postinjection, there were no significant differences in tumor volume between CL1-0 Vec tumors and CL1-0 AK4 tumors (Fig. 5a). However, by examining pimonidazole staining, we surprisingly found that the hypoxic necrosis tumor area was significantly reduced in CL1-0 AK4 tumors compared with CL1-0 Vec tumors (Fig. 5b).We further performed IHC analysis of serial sections from CL1-0 AK4 and CL1-0 Vec xenograft tumors, and the results revealed a strong positive correlation between AK4 expression and nuclear HIF-1α levels and a negative correlation between AK4 and E-cadherin expression in vivo (Fig. 5c). To further determine the effect of AK4 overexpression on metastasis, we injected AK4-expressing and vector-expressing CL1-0 cells into the left lung of NSG mice. Four weeks postinjection, we found that overexpression of AK4 significantly promoted CL1-0 cell metastasis to the liver (Fig. 5d). Quantification of the metastatic nodules using H&E staining and histological examination confirmed that the number of nodules in the liver was significantly increased in mice carrying AK4-overexpressing tumors compared with mice harboring vector-expressing tumors (Fig. 5e).

### Identification of novel drug candidates for metastatic lung cancer through querying pharmacogenomics profiles of the AK4 gene signature

**Fig. 6:**
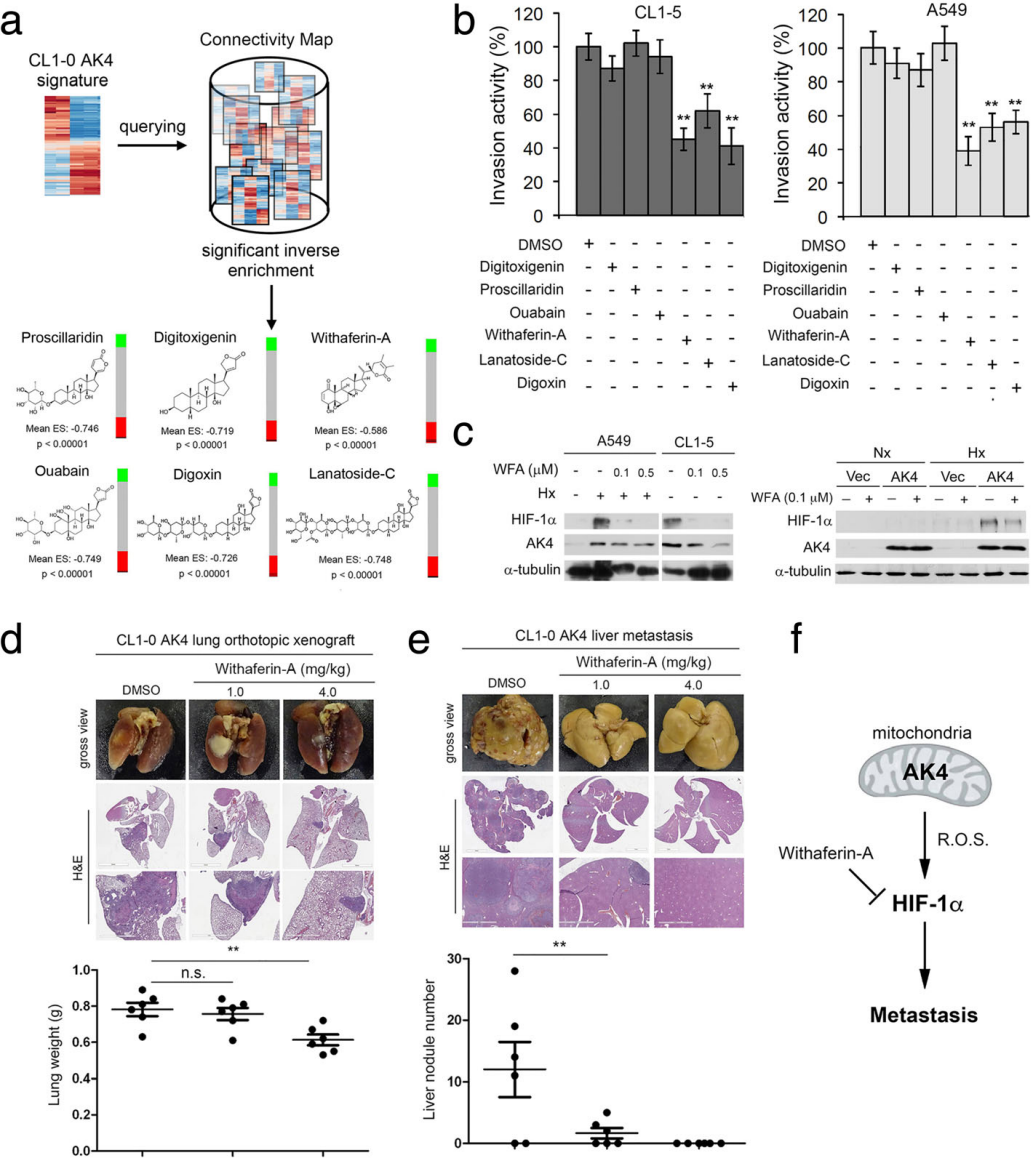
Connectivity map analysis of AK4 gene signature identifies withaferin-A as a potent anti-metastatic agent for NSCLC. **a** Identification of structurally similar drug candidates with the best reverse AK4 gene expression enrichment score by querying the connectivity map. **b** Invasion assay of A549 and CL1-5 cells treated with the corresponding IC10 doses of drug candidates. The data are expressed as percent inhibition compared with DMSO as the vehicle control. **P ≤ 0.01 **c** WB analysis of HIF-1α and AK4 protein levels in A549, CL1-5, CL1-0 vector-, and AK4-expressing cells treated with or without withaferin-A under Nx or Hx. **d** Gross view (formalin-fixed) and H&E staining images of lungs from mice treated with DMSO vehicle control or withaferin-A (1.0 mg/kg or 4.0 mg/kg) at day 30 after orthotopic injection of CL1-0 cells overexpressing AK4 (top). Quantification of tumor weight in lungs of mice treated with DMSO vehicle control or withaferin-A (1.0 mg/kg or 4.0 mg/kg) at day 28 after orthotopic injection of CL1-0 cells overexpressing AK4 (bottom). **e** Gross view (formalin-fixed) and H&E staining images of livers from mice treated with DMSO vehicle control or withaferin-A (1.0 mg/kg or 4.0 mg/kg) at day 30 after orthotopic injection of CL1-0 cells overexpressing AK4 (top). Quantification of liver nodule number in mice treated with DMSO vehicle control or withaferin-A (1.0 mg/kg or 4.0 mg/kg) at day 30 after orthotopic injection of CL1-0 cells overexpressing AK4 (bottom). **f** Diagram depicting a working model of AK4-induced HIF-1α stabilization via intracellular ROS elevation, leading to subsequent EMT and metastasis. Targeting of the AK4-HIF-1α axis by withaferin-A impairs lung cancer metastasis. The results are presented as the mean ± SD of at least three separate experiments. Two-tailed, unpaired Student’s *t* tests were used for all pairwise comparisons. *P ≤ 0.05; **P ≤ 0.01

To identify drug candidates that could reverse the AK4 gene expression profile as a therapeutic strategy to inhibit lung cancer metastasis, we queried a connectivity map using differentially expressed genes upon AK4 overexpression in CL1-0 cells and identified six structurally similar candidate drugs that showed the best enrichment scores, namely, proscillaridin, ouabain, digitoxigenin, digoxin, withaferin-A, and lanatoside-C (Fig. 6a). We then conducted MTT cell viability assays after exposure to the drugs in culture and determined the IC_50_ and IC_10_ for each of the drugs in CL1-0, CL15, CL1-0 Vec, and CL1-0 AK4 cells (Additional file 1: Figure S4). Furthermore, we screened the anti-invasion effect of each drug at the IC_10_ dose in A549 and CL1-5 cells and found that withaferin-A, lanatoside-C, and Digoxin significantly suppressed the invasion ability of A549 and CL1-5 cells (Fig. 6b). Interestingly, digoxin and lanatoside-C were shown to be potent HIF-1 modulators, while the effect of withaferin-A (WFA) on modulating HIF-1 α and AK4 levels was not clear. We then treated A549, CL1-5, CL1-0 Vec, and CL1-0 AK4 cells with WFA under Nx or Hx, and WB analysis showed that HIF-1α and AK4 protein levels were significantly downregulated upon WFA treatment (Fig. 6c). To evaluate the anti-metastasis effect of WFA, we orthotopically injected CL1-0 AK4 cells into the left lung of NSG mice, and the mice were subsequently treated with WFA at a dose of 1.0 mg/kg and 4.0 mg/kg three times per week through intraperitoneal administration. At day 28, the lungs and livers were removed for pathological examination. The quantitative liver nodule number data confirmed the anti-metastasis effect of WFA because mice who received WFA at 1.0 mg/kg showed a significant reduction in liver metastatic nodule number (Fig. 6d, e). In mice who received WFA at 4.0 mg/kg, the data showed a promising therapeutic effect of suppressing both primary and metastatic tumors (Fig. 6d, e). Similar anti-metastasis effects of WFA were also observed in treating A549 orthotopic lung cancer model mice; WFA at 1.0 mg/kg induced significant inhibition of liver metastasis, while mice who received WFA at 4.0 mg/kg showed the lowest lung tumor burden, with no sign of distal metastasis (Additional file 1: Figure S5). Taken together, our data suggest that the AK4-HIF-1α signaling axis is a potential therapeutic target of lung cancer metastasis and WFA might be a potential compound for further development to treat metastatic lung cancer (Fig. 6f).

## Discussion

Dysregulation of HIF is increasingly recognized as a critical step during cancer progression [19]. Deletion of *Hif1a* has been reported to markedly impair metastasis in a mouse mammary tumor virus (MMTV) promoter-driven polyoma middle T antigen mouse model of breast cancer [20]. In an orthotopic xenograft model of lung cancer, the HIF-1α antagonist PX-478 effectively inhibits tumor progression [21]. Here, we identified a novel signaling axis whereby enhanced expression of AK4 exaggerates HIF-1α protein expression, leading to EMT induction in lung cancer. Previous studies have shown that AK4 is one of the hypoxia responsive genes and AK4 is also a transcriptional target of HIF-1α [22–25]. Surprisingly, we found that the presence of AK4 can exert feedback regulation of HIF-1α and this AK4-HIF-1α positive feedback loop operates through elevation of intracellular ROS levels, which stabilize HIF-1α protein and induce EMT.

In lung cancer, both small cell and non–small cell lung cancers express high levels of HIF-1α, but its role as a prognostic factor remains controversial. HIF-1α expression has been reported as a poor prognosis marker in several studies [26–29]. However, some studies reported inconsistent results showing that the predictive power of HIF-1α as a prognosis marker is only marginal [30–32]. In our study, patients with high levels of HIF-1α showed a trend toward poor prognosis compared with those exhibiting low levels of HIF-1α. Furthermore, we found that the combined AK4 and HIF-1α status could significantly augment the prognostic power compared with HIF-1α alone (Fig. 2c). Taken together, our results suggest that AK4 may serve as a critical factor dictating the prognostic power of HIF-1α in lung cancer patients.

It is widely known that HIF-1α regulation mainly occurs at the level of protein stability. Under normoxic conditions, HIF-1α is hydroxylated at two conserved proline residues (P402 and P577) by a family of HIF prolyl hydroxylase enzymes that includes PHD1, PHD2, and PHD3. Hydroxylated HIF-1α is then polyubiquitinated by E3 ubiquitin ligase, leading to proteosomal degradation. In hypoxia, hydroxylation does not occur due to the lack of substrate oxygen for PHDs. Moreover, ROS have been reported to inhibit the activity of PHDs [33–36]. In our study, overexpression of AK4 reduced HIF-1α hydroxylation in the presence of MG132, suggesting that AK4 may stabilize HIF-1α protein by decreasing PHD activity via ROS accumulation. Although the mechanism of AK4-mediated ROS production is unclear, the subcellular localization and physiological function of AK4 may provide a clue to the possible mechanism. AK4 interacts with ANT and voltage-dependent anion channel (VDAC) at the mitochondrial matrix, and their interactions are required for regulation of mitochondria membrane permeability and export of ATP from the mitochondrial matrix to the cytosol in exchange for ADP import from the cytosol to the mitochondrial matrix [9]. Thus, AK4 may regulate the efflux of ROS generated from the electron transport chain (ETC) to the cytosol by interacting with the ANT/VDAC complex. Our results are consistent with those of other studies suggesting that ROS generated from the ETC could contribute to HIF-1α stabilization by blocking HIF-1α hydroxylation and von Hippel Lindau (pVHL) protein binding [37–39].

Prior studies have reported that hypoxia or overexpression of HIF-1α can induce EMT through direct binding of HIF-1α to the hypoxia response elements (HREs) within the Snail and Twist promoters [40, 41]. Other EMT regulators, such as Zeb1, Zeb2, and TCF-3, have been reported to be upregulated in pVHL-null renal cell carcinoma in which HIF-1α is constitutively overexpressed [42]. In addition to binding to the canonical HRE, HIF-1α can also interact with a variety of co-factors to activate EMT-associated genes and diverse gene expression in response to hypoxia [43, 44].

Tumors rewire metabolism to provide sufficient energy and biosynthetic intermediates to meet the requirements of uncontrolled proliferation and progression. Enhanced glucose metabolism not only produces energy but also provides macromolecular precursors and maintains NADPH homeostasis for cancer cells to withstand oxidative stress [45]. A recent study suggested that increased ROS production is essential to enable and sustain a metastatic phenotype [46]. However, large-scale clinical trials using an antioxidant supplement as a preventive and therapeutic anticancer strategy did not show a beneficial effect in cancer patients. In contrast, an antioxidant supplement even increased tumor incidence in a genetically engineered mouse model of lung cancer and melanoma [47, 48]. One possible explanation for these controversial results is that cancer cells adapt to have a tight redox regulation system that allows them to withstand higher ROS accumulation than normal cells but below a critical cytotoxic threshold. The use of general antioxidants might alleviate circulating tumor cells from oxidative stress and accelerate metastasis development. Therefore, a buildup oxidative stress and being equipped with antioxidant defenses is critical for tumors to metastasize [49]. Through ingenuity upstream regulator analysis of the AK4 metabolic signature, we also found that NRF2, the master regulator of antioxidant responses, was significantly activated, suggesting that high AK4 expression lung adenocarcinoma patients may be accompanied by NRF2 activation (Fig. 1c). Furthermore, microarray analysis revealed that genes encoding enzymes in the glutathione metabolism pathway were differentially expressed upon AK4 overexpression in CL1-0 cells (Fig. 4b and Additional file 1: Figure S3B). Moreover, in animal studies, we showed that overexpression of AK4 not only protects tumors from hypoxic necrosis but also enhances their ability to metastasize. These data are consistent with the notion that only cancer cells equipped with an enhanced antioxidant defense system are capable of leveraging oxidative stress to promote metastasis. Our findings suggest that overexpression of AK4 may trigger metabolic adaptation toward increased intracellular oxidative stress and antioxidant capacity at the same time and subsequently promote HIF-1α-mediated EMT and metastatic dissemination.

Dysregulation of E-cadherin protein through post-translational glycosylation has been shown to be a critical event during cancer progression [50]. Specifically, the modification of E-cadherin protein at Asn-554 with β1,6-*N*-acetylglucosamin (β1,6GlcNAc)-branched *N*-glycan catalyzed by *N*-acetylglucosaminyltransferase V (GnT-V) disrupts its cell adhesion function and therefore enhances tumor invasion [51, 52]. In the AK4 metabolic gene signature, we identified genes encode for enzymes in *N*-glycan, mucin type *O*-glycan, and glycosaminoglycan biosynthesis pathways were significantly enriched (Fig. 1b). To this end, we also found the expression GnT-V protein was regulated by AK4 under hypoxia in a HIF-1α-dependent manner (Additional file 1: Figure S2A). However, the impact of AK4 expression and/or hypoxia on global glycosylation profile in lung cancer remains to be further elucidated.

Through pharmacogenomics analysis, we identified withaferin-A as a potential inhibitor that reverses the AK4-induced gene signature and acts as a potent anti-metastatic agent in lung cancer. Similar to our findings, Hahm et al. showed that withaferin-A treatment inhibits lung metastasis by suppressing glycolysis in a mouse mammary tumor virus–neu (MMTV-neu) transgenic model, which highlights the therapeutic opportunities for targeting the metabolic vulnerability of tumors [53].

In conclusion, we suggest that overexpression of AK4 stabilizes HIF-1α protein by increasing intracellular ROS levels and induces EMT in NSCLC. More importantly, pharmacologically reversing the AK4 gene signature (e.g., with withaferin-A) may serve as an effective strategy to treat metastatic lung cancer.

## Additional file

Additional file 1: **Table S1.** Correlation of clinicopathological features of NSCLC patients with AK4 and HIF-1α expression. **Figure S1.** (related to Fig. 1) Ingenuity upstream analysis of consensus AK4 metabolic gene signature between GSE31210 and TCGA LUAD. A, Venn diagram analysis of AK4 metabolic gene signature in GSE31210 and TCGA LUAD datasets. Activation *z* score more than 2 or less than − 2 is predicted to be significant activation or inhibition respectively. B, Left panel, Ingenuity upstream analysis of consensus AK4 metabolic signature. Right panel, heatmap illustrates HIF-1 α -regulated genes that are positively or negatively correlated with AK4 expression in consensus AK4 metabolic signature. **Figure S2.** (related to Fig. 3) AK4-induced EMT is HIF-1α-dependent. A, WB analysis of AK4, HIF-1α, GnT-V, E-cadherin, Vimentin, Snail from CL1-0 vector- or AK4-expressing cells transduced with shNS or shHIF-1α in Hx.B, Invasion assay of CL1-0 vector- or AK4-expressing cell transduced with shNS or shHIF-1α in Hx. The results are presented as the mean ± SD of at least three separate experiments. Two-tailed, unpaired Student’s *t* tests were used for all pairwise comparisons. *P ≤ 0.05; **P ≤ 0.01. **Figure S3.** (related to Fig. 4) Differentially expressed genes in glycolysis/gluconeogenesis and glutathione metabolism in CL1-0 upon AK4 overexpression. A, Relative expression level of genes in KEGG glycolysis and gluconeogenesis pathway from CL1-0 AK4 versus CL1-0 Vec microarray data. B, Relative expression level of genes in KEGG glutathione metabolism pathway from CL1-0 AK4 versus CL1-0 Vec microarray data. **Figure S4.** (related to Fig. 6) MTT assay cell viability assay of digitoxigenin, lanatoside C, digoxin, proscillaridin, and withaferin-A in CL1-0, CL1-5, CL1-0 Vec, and CL1-0 AK4. **Figure S5.** (related to Fig. 6) Withaferin-A treatment suppresses metastasis in A549 orthotopic lung cancer mouse model. A, 5 A549-GL cells were orthotopically injected into the left lung of NSG mice that were treated over an interval of one day with DMSO vehicle control or withaferin-A: 1.0 mg/kg; 4.0 mg/kg. Luminescence was measured using a noninvasive, bioluminescence imaging system (IVIS spectrum) at days 1 (top) and 28 (bottom). B, Luminescence, fluorescence, gross view (formalin-fixed) and H&E staining images in the lungs of mice treated with DMSO vehicle control or withaferin-A (1.0 mg/kg or 4.0 mg/kg) at day 28 after orthotopic injection of A549-GL cells (top). Quantification of tumor weight in the lung of mice treated with DMSO vehicle control or withaferin-A (1.0 mg/kg or 4.0 mg/kg) at day 28 after orthotopic injection of A549-GL cells (bottom). C, Luminescence, fluorescence, gross view (formalin-fixed) and H&E staining images in the livers of mice treated with DMSO vehicle control or withaferin-A (1.0 mg/kg or 4.0 mg/kg) at day 28 after orthotopic injection of A549-GL cells (top). Quantification of liver nodule number in the mice treated with DMSO vehicle control or withaferin-A (1.0 mg/kg or 4.0 mg/kg) at day 28 after orthotopic injection of A549-GL cells (bottom). The results are presented as the mean ± SD of at least three separate experiments. Two-tailed, unpaired Student’s *t* tests were used for all pairwise comparisons. *P ≤ 0.05; **P ≤ 0.01. (PDF 3400 kb)

## Abbreviations

AK4: Adenylate kinase 4
EMT: Epithelial-to-mesenchymal transition
HIF-1α: Hypoxia-inducible factor 1-alpha
NAC: *N*-acetylcysteine
ROS: Reactive oxygen species
WFA: Withaferin-A
