# Supplementary material for: Adenylate kinase 4 modulates oxidative stress and stabilizes HIF-1α to drive lung adenocarcinoma metastasis
Source: J Hematol Oncol. 2019 Jan 29;12:12. doi: 10.1186/s13045-019-0698-5 (PMC6352453; doi:10.1186/s13045-019-0698-5)

Supplemental Information

Adenylate kinase-4 modulates oxidative stress and stabilizes HIF-1α to drive lung adenocarcinoma metastasis

Yi-Hua Jan[1], Tsung-Ching Lai[1], Chih-Jen Yang[2], Yuan-Feng Lin[3], Ming-Shyan Huang[2*], and Michael Hsiao[1,4*]

[1]Genomics Research Center, Academia Sinica, Taipei, Taiwan, [2]Department of Internal Medicine, Kaohsiung Medical University Hospital, School of Medicine, Kaohsiung Medical University, Kaohsiung, Taiwan, [3]Graduate Institute of Clinical Medicine, College of Medicine, Taipei Medical University, Taipei, Taiwan. [4]Department of Biochemistry, College of Medicine, Kaohsiung Medical University, Kaohsiung, Taiwan

***Correspondence to:** Dr. Michael Hsiao, Genomics Research Center, Academic Sinica, 128 Academia Road, Section 2, Taipei 115, Taiwan. Phone: 886-2-2787-1243; Fax: 886-2-2789-9931; E-mail: mhsiao@gate.sinica.edu.tw

Supplemental Data

Figure S1	Related to Figure 1
Figure S2	Related to Figure 3
Figure S3	Related to Figure 4
Figure S4	Related to Figure 6
Figure S5	Related to Figure 6

Table S1. Correlation of clinicopathological features of NSCLC patients with AK4 and HIF-1α expression

Clinicopathological feature	n	AK4 expression, n (%)		*P*	HIF-1α expression, n (%)		*P*
		Low (n=42)	High (n=58)		Low (n=21)	High (n=79)	
Age							
<65 y	55	27 (49.1)	28 (50.9)	0.112	14 (25.5)	41 (74.5)	0.226
≧65 y	45	15 (33.3)	30 (66.7)		7 (15.6)	38 (84.4)	
Gender							
Male	54	22 (40.7)	32 (59.3)	0.782	9 (16.7)	45 (83.3)	0.249
Female	46	20 (43.5)	26 (56.5)		12 (26.1)	34 (73.9)	
Smoking							
Non-smoker	62	27 (43.5)	35 (56.5)	0.856	11 (17.7)	51 (82.3)	0.306
Smoker	38	15 (39.5)	23 (60.5)		10 (26.3)	28 (73.7)	
Histology							
Adenocarcinoma	62	29 (46.8)	33 (53.2)	0.408	18 (29.0)	44 (71.0)	N.A.
Squamous cell carcinoma	31	10 (32.3)	21 (67.7)		3 (9.7)	28 (90.3)	
Large cell carcinoma	7	3 (42.9)	4 (57.1)		0 (0.0)	7 (100.0)	
T stage							
T1+T2	68	26 (38.2)	42 (61.8)	0.266	12 (17.6)	56 (82.4)	0.230
T3+T4	32	16 (50.0)	16 (50.0)		9 (28.1)	23 (71.9)	
N stage							
N0	36	22 (61.1)	14 (38.9)	**0.003**	12 (33.3)	24 (66.7)	**0.023**
N1-N3	64	20 (31.3)	44 (68.7)		9 (14.1)	55 (85.9)	
M stage							
M0	71	32 (45.1)	39 (54.9)	0.330	14 (19.7)	57 (80.3)	0.622
M1	29	10 (34.5)	19 (65.5)		7 (24.1)	22 (75.9)	
Pathological stage							
I + II	42	21 (50.0)	21 (50.0)	0.167	10 (28.6)	32 (71.4)	0.557
III + IV	58	21 (36.2)	37 (63.8)		11 (19.0)	47 (81.0)	
Recurrence							
No	23	12 (52.2)	11 (47.8)	0.259	6 (26.1)	17 (73.9)	0.494
Yes	77	30 (39.0)	47 (61.0)		15 (19.5)	62 (80.5)	

P value < 0.05 was considered statistically significant (Pearson chi-square test for categorical variables). The tumor stage, tumor, lymph node, and distal metastasis status were classified according to the international system for staging lung cancer.

Supplemental Figure Legends

Supplementary Fig. S1 (related to Figure 1) Ingenuity upstream analysis of consensus AK4 metabolic gene signature between GSE31210 and TCGA LUAD. **A**, Venn diagram analysis of AK4 metabolic gene signature in GSE31210 and TCGA LUAD datasets. Activation Z-score more than 2 or less than -2 is predicted to be significant activation or inhibition respectively. **B**, Left panel, Ingenuity upstream analysis of consensus AK4 metabolic signature. Right panel, heatmap illustrates HIF-1 α -regulated genes that are positively or negatively correlated with AK4 expression in consensus AK4 metabolic signature.

Supplementary Fig. S2 (related to Figure 3) AK4-induced EMT is HIF-1α-dependent. **A**, WB analysis of AK4, HIF-1α, GnT-V, E-cadherin, Vimentin, Snail from CL1-0 vector- or AK4-expressing cells transduced with shNS or shHIF-1α in Hx.**B**, Invasion assay of CL1-0 vector- or AK4-expressing cell transduced with shNS or shHIF-1α in Hx. The results are presented as the mean ± SD of at least three separate experiments. Two-tailed, unpaired Student's t-tests were used for all pairwise comparisons. **P* ≤ 0.05; ***P* ≤ 0.01

Supplementary Fig. S3 (related to Figure 4) Differentially expressed genes in glycolysis/gluconeogenesis and glutathione metabolism in CL1-0 upon AK4 overexpression. **A**, Relative expression level of genes in KEGG glycolysis and gluconeogenesis pathway from CL1-0 AK4 versus CL1-0 Vec microarray data. **B**, Relative expression level of genes in KEGG glutathione metabolism pathway from CL1-0 AK4 versus CL1-0 Vec microarray data.

Supplementary Fig. S4 (related to Figure 6) MTT assay cell viability assay of digitoxigenin, lanatoside C, digoxin, proscillaridin, and withaferin-A in CL1-0, CL1-5, CL1-0 Vec and CL1-0 AK4

Supplementary Fig. S5 (related to Figure 6) Withaferin-A treatment suppresses metastasis in A549 orthotopic lung cancer mouse model. **A**,

A549-GL cells were orthotopically injected into the left lung of NSG mice that were treated over an interval of one day with DMSO vehicle control or Withaferin-A: 1.0 mg/kg; 4.0 mg/kg. Luminescence was measured using a noninvasive, bioluminescence imaging system (IVIS spectrum) at days 1 (*top*) and 28 (*bottom*). **B**, Luminescence, fluorescence, gross view (formalin-fixed) and H&E staining images in the lungs of mice treated with DMSO vehicle control or Withaferin-A (1.0 mg/kg or 4.0 mg/kg) at day 28 after orthotopic injection of A549-GL cells (*top*). Quantification of tumor weight in the lung of mice treated with DMSO vehicle control or Withaferin-A (1.0 mg/kg or 4.0 mg/kg) at day 28 after orthotopic injection of A549-GL cells (*bottom*). **C**, Luminescence, fluorescence, gross view (formalin-fixed) and H&E staining images in the livers of mice treated with DMSO vehicle control or Withaferin-A (1.0 mg/kg or 4.0 mg/kg) at day 28 after orthotopic injection of A549-GL cells (*top*). Quantification of liver nodule number in the mice treated with DMSO vehicle control or Withaferin-A (1.0 mg/kg or 4.0 mg/kg) at day 28 after orthotopic injection of A549-GL cells (*bottom*). The results are presented as the mean ± SD of at least three separate experiments. Two-tailed, unpaired Student's t-tests were used for all pairwise comparisons. *$P \leq 0.05$; **$P \leq 0.01$

Fig. S1 (Jan *et al.*)

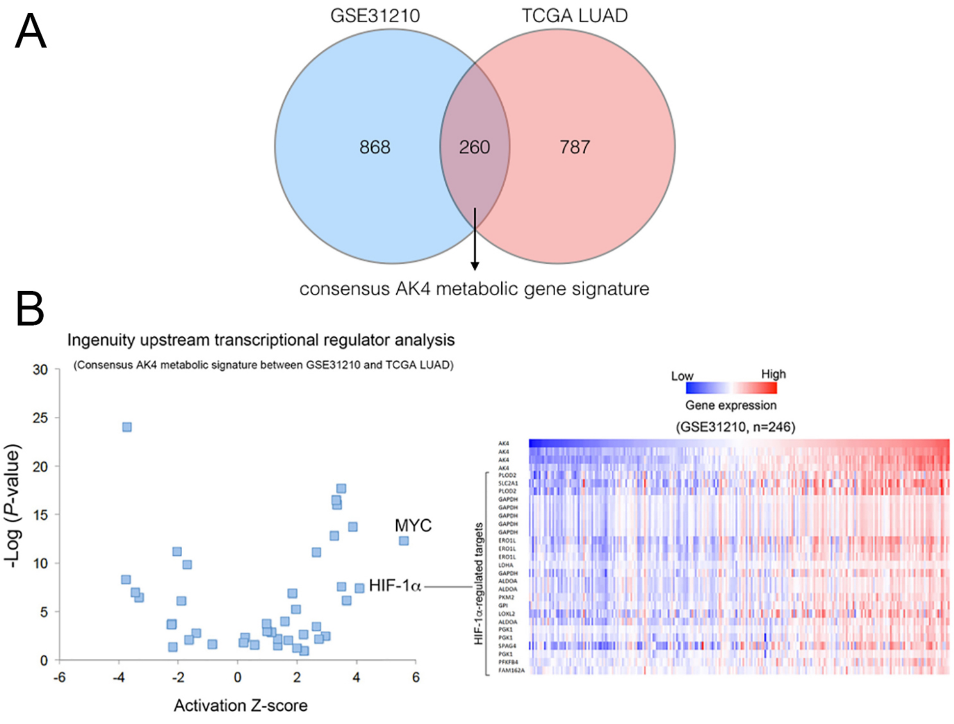

Fig. S2 (Jan *et al*.)

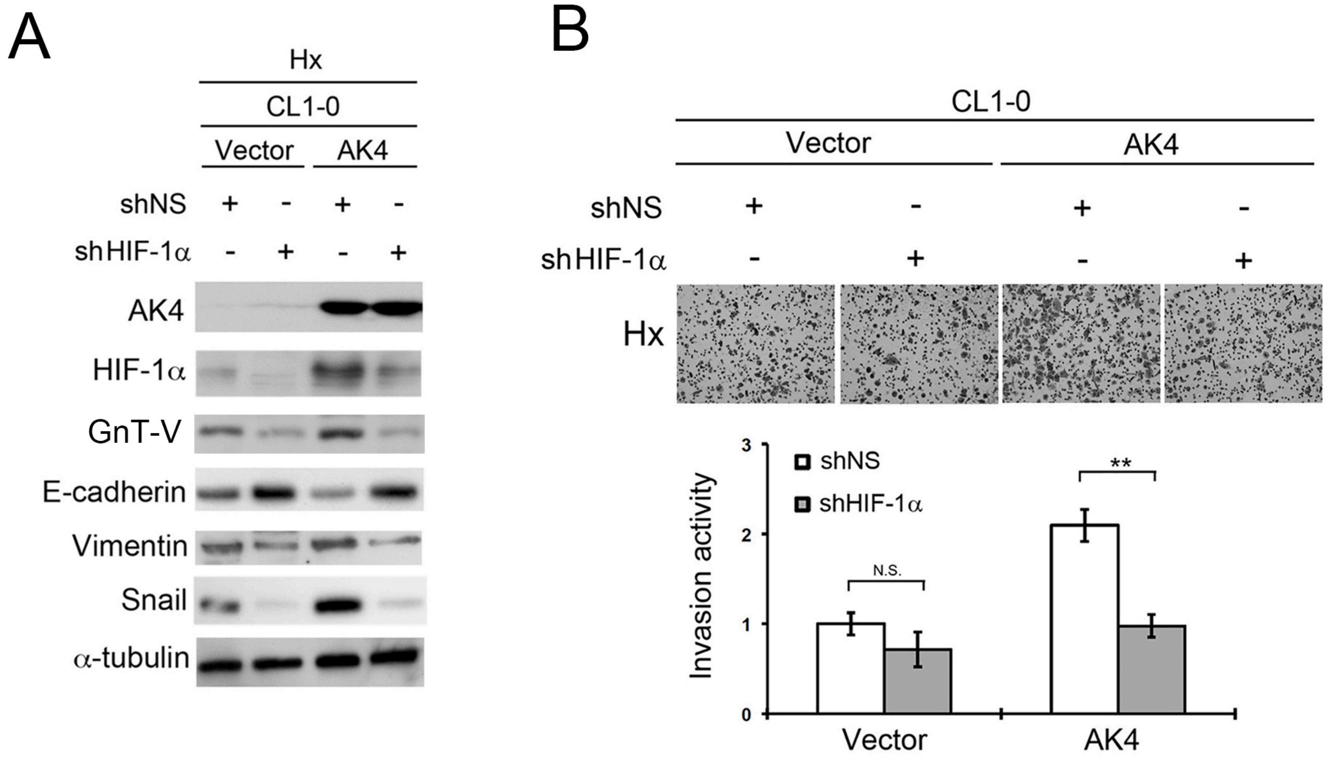

Fig. S3 (Jan *et al.*)

A

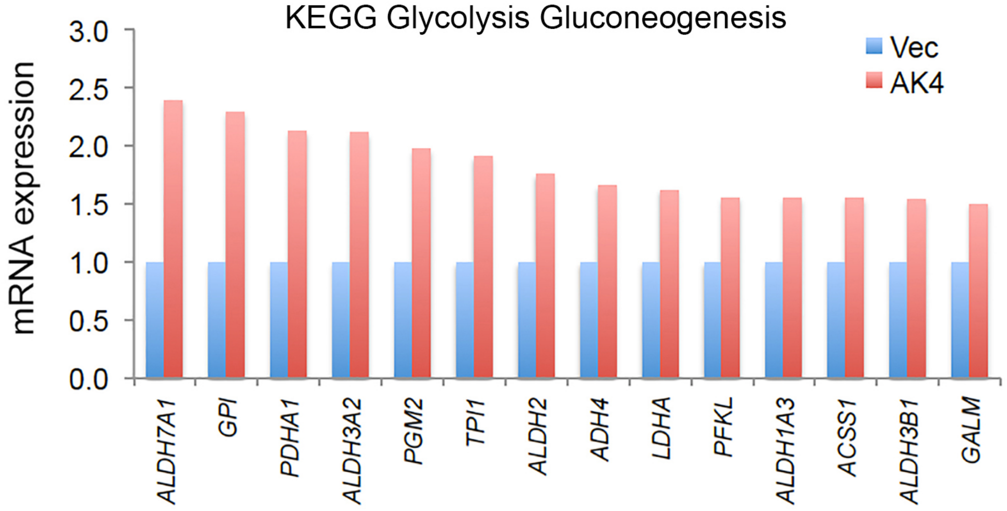

B

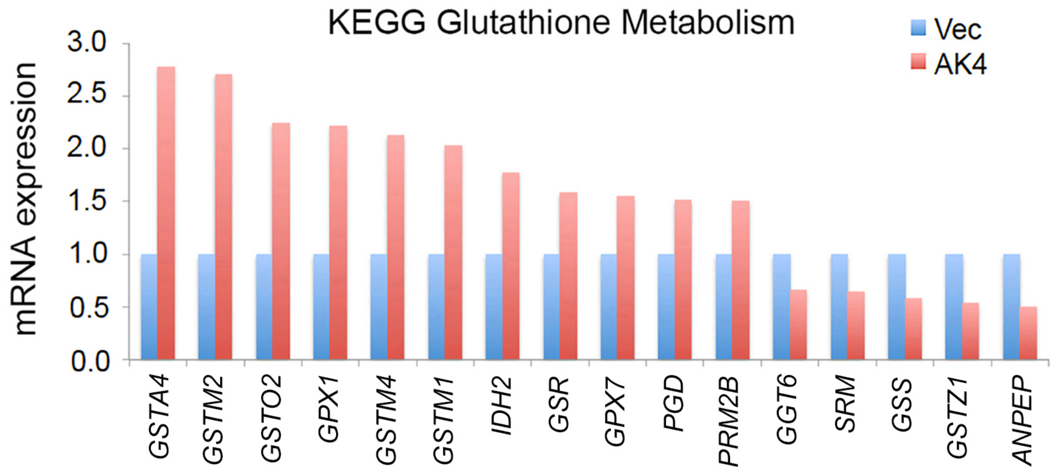

Fig.S4 (Jan *et al*.)

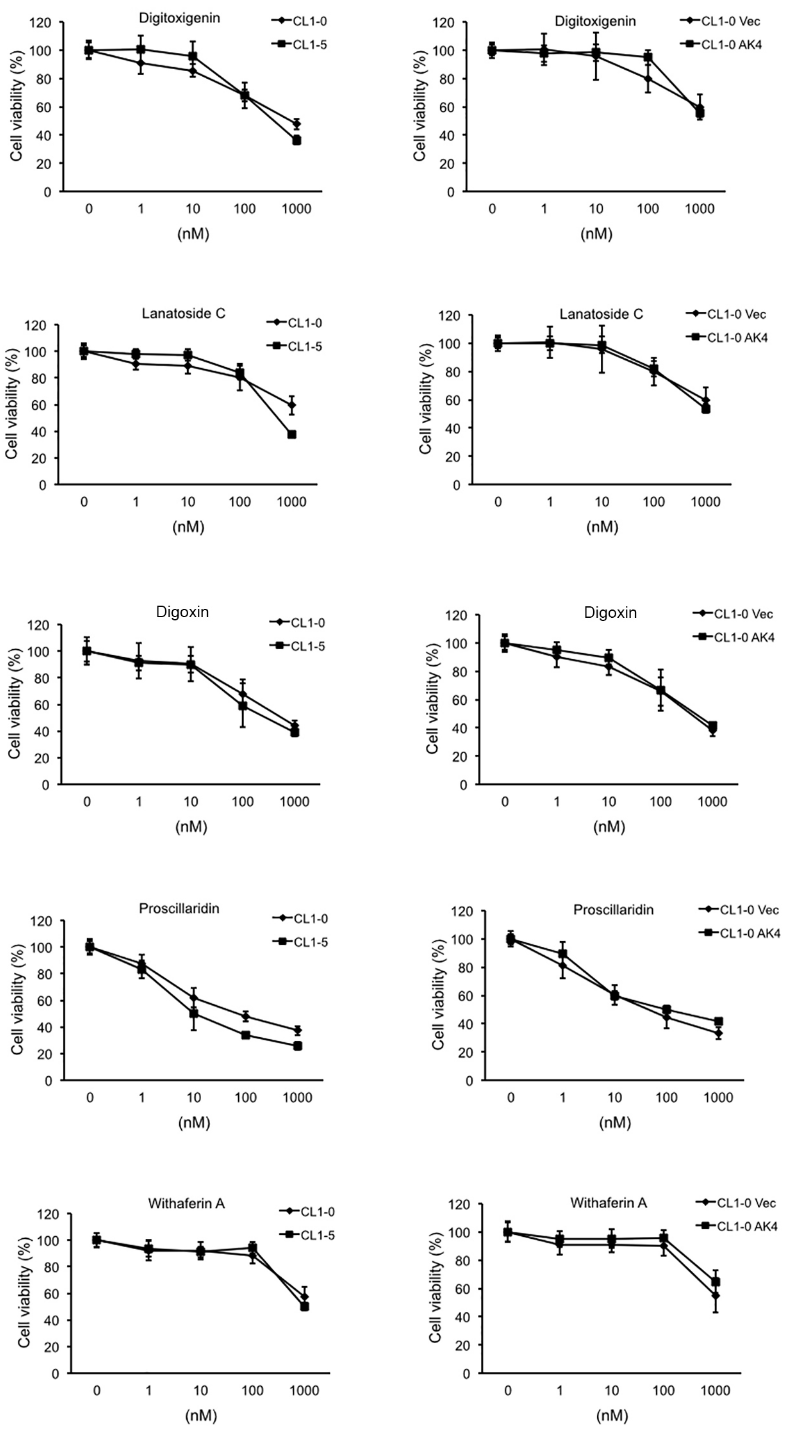

Fig. S5 (Jan *et al.*)

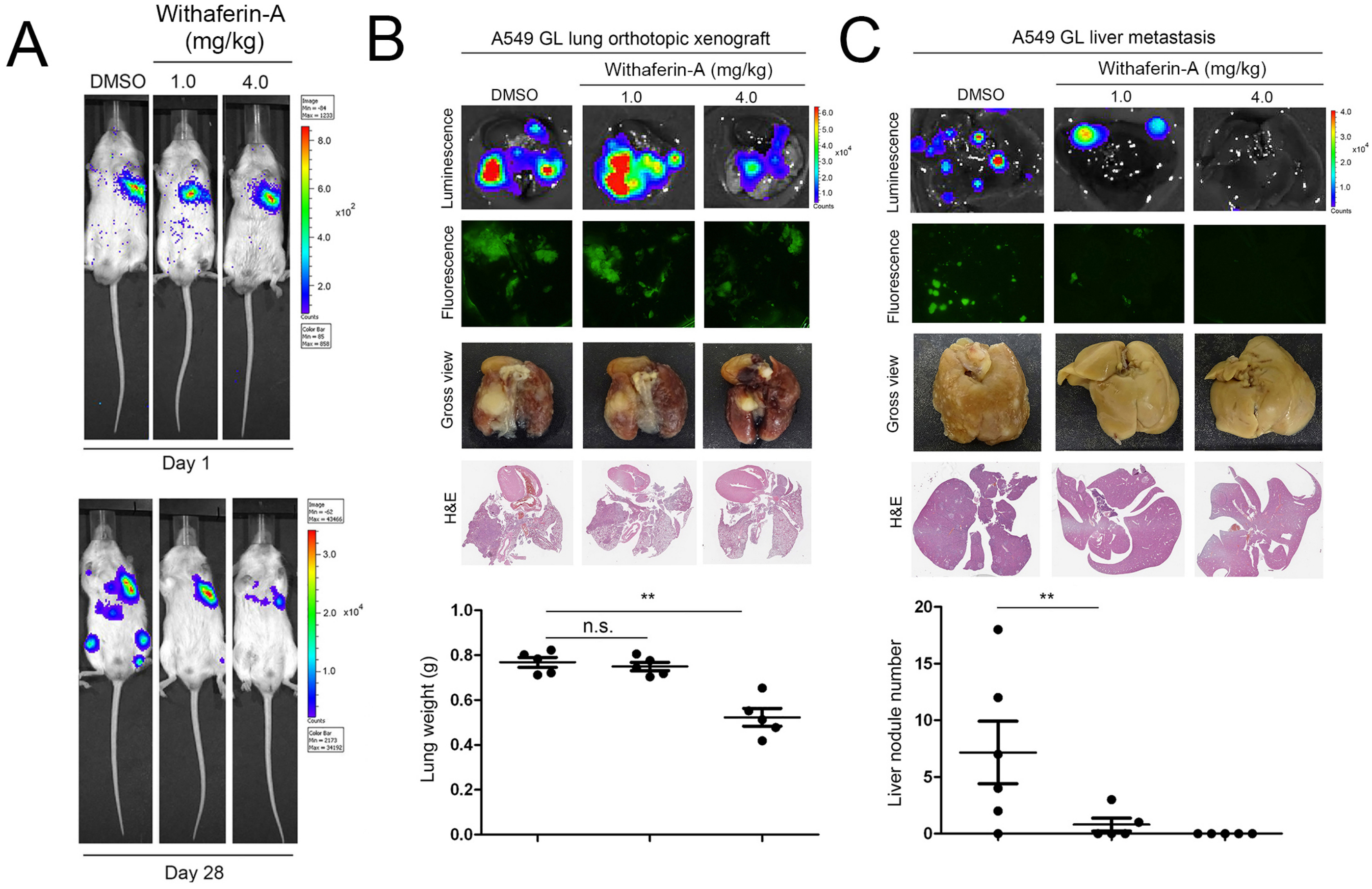

Supplement: Supplementary file 1 — Table S1. Correlation of clinicopathological features of NSCLC patients with AK4 and HIF-1α expression. Figure S1. (related to Fig. 1) Ingenuity upstream analysis of consensus AK4 metabolic gene signature between GSE31210 and TCGA LUAD. A, Venn diagram analysis of AK4 metabolic gene signature in GSE31210 and TCGA LUAD datasets. Activation z score more than 2 or less than − 2 is predicted to be significant activation or inhibition respectively. B, Left panel, Ingenuity upstream analysis of consensus AK4 metabolic signature. Right panel, heatmap illustrates HIF-1 α -regulated genes that are positively or negatively correlated with AK4 expression in consensus AK4 metabolic signature. Figure S2. (related to Fig. 3) AK4-induced EMT is HIF-1α-dependent. A, WB analysis of AK4, HIF-1α, GnT-V, E-cadherin, Vimentin, Snail from CL1-0 vector- or AK4-expressing cells transduced with shNS or shHIF-1α in Hx.B, Invasion assay of CL1-0 vector- or AK4-expressing cell transduced with shNS or shHIF-1α in Hx. The results are presented as the mean ± SD of at least three separate experiments. Two-tailed, unpaired Student’s t tests were used for all pairwise comparisons. *P ≤ 0.05; **P ≤ 0.01. Figure S3. (related to Fig. 4) Differentially expressed genes in glycolysis/gluconeogenesis and glutathione metabolism in CL1-0 upon AK4 overexpression. A, Relative expression level of genes in KEGG glycolysis and gluconeogenesis pathway from CL1-0 AK4 versus CL1-0 Vec microarray data. B, Relative expression level of genes in KEGG glutathione metabolism pathway from CL1-0 AK4 versus CL1-0 Vec microarray data. Figure S4. (related to Fig. 6) MTT assay cell viability assay of digitoxigenin, lanatoside C, digoxin, proscillaridin, and withaferin-A in CL1-0, CL1-5, CL1-0 Vec, and CL1-0 AK4. Figure S5. (related to Fig. 6) Withaferin-A treatment suppresses metastasis in A549 orthotopic lung cancer mouse model. A, 5 A549-GL cells were orthotopically injected into the left lung of NSG mice that were treated ov [file 13045_2019_698_MOESM1_ESM.pdf]
